# Use of the comet assay technique for quick and reliable prediction of *in vitro* response to chemotherapeutics in breast and colon cancer

**DOI:** 10.1186/2241-5793-21-14

**Published:** 2014-08-01

**Authors:** Panagiotis Apostolou, Maria Toloudi, Eleni Kourtidou, Georgia Mimikakou, Ioanna Vlachou, Marina Chatziioannou, Ioannis Papasotiriou

**Affiliations:** Research Genetic Cancer Centre Ltd (R.G.C.C. Ltd), Filotas, Florina Greece

**Keywords:** Comet assay, Personalized medicine, Breast cancer, Colon cancer, Cisplatin, Melphalan, Mechlorethamine, Doxorubicin

## Abstract

**Background:**

Determination of response to chemotherapy is a major requirement of personalized medicine. Resistance, whether developed or native, critically affects a treatment’s success. Single Cell Gel lectrophoresis - also known as a comet assay - is used to detect DNA damage at the level of individual eukaryotic cells. We assessed the use of comet assays in determining response to chemotherapeutic drugs that are widely used in breast and colon cancer.

**Results:**

We treated human breast and colon cancer cell lines with melphalan, cisplatin, mechlorethamine or doxorubicin, as monotherapies. Drug activities varied even in the same cancer types, further demonstrating the heterogeneity of different cancer types.

**Conclusion:**

The comet assay technique can provide reliable and quick results with minimum requirements and is applicable to a wide variety of drugs.

## Background

Personalized medicine requires that therapy should be customized to individual patients, using genetic or other information [1]. Prediction of response to chemotherapy drugs is a major concern in cancer treatment [2]. Resistance to chemotherapy agents may exist before, or develop during, therapy [3]. Most techniques to predict response entail analysis of expression of different genes [4]. However, fast, reliable results are urgently needed; moreover, a drug’s effect cannot always be predicted by measuring gene expression [5].

Single Cell Gel Electrophoresis (SCGE), also known as comet assay can measure DNA damage in individual eukaryotic cells. The principle of the comet assay is that unfragmented DNA maintains a well-organized structure in the nucleus, but becomes disrupted when the cell is damaged. It detects both single-strand and double-strand breaks, and has a simple and inexpensive setup. Comet assay is therefore a promising technique for predicting response to drugs that are affected by DNA structure [6].

The present study evaluated the predictability of response to widely used chemotherapy drug from comet assay results in established human breast and colon cancer cell lines. We tested nitrogen-mustard alkylating agents (melphalan, mechlorethamine) and doxorubicin in breast cancer, and tested cisplatin in colon cancer cell lines.

## Results and discussion

**Figure 1:**
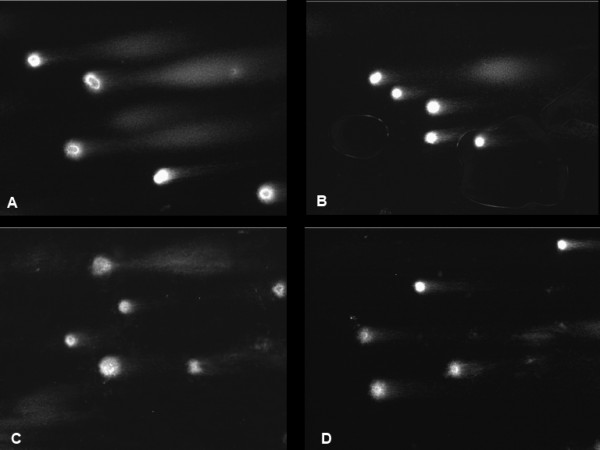
**Comet assay data for MDA-MB 231 cell line. A**: Untreated cell line after alkaline lysis. **B**: The percentage of DNA in tail has been reduced after incubation with doxorubicin. **C**: Data after incubation with melphalan. **D**: Treatment with mechlorethamine.

**Figure 2:**
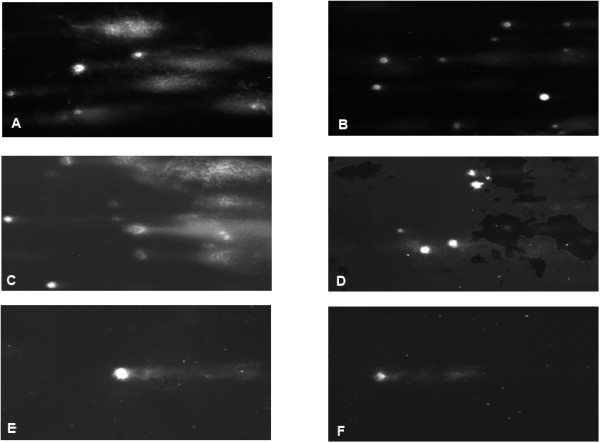
**Comet assay data for cancer cell lines representing colorectal cancer. A**: Control HCT-15 cell line without cisplatin. **B**: HCT-15 after incubation with cisplatin. **C**: Untreated HCT-116 cell line. **D**: HCT-116 with cisplatin. **E**: LoVo cancer cell line without addition of drugs. **F**: The percentage of DNA in tail is reduced after cisplatin incubation; however the data are not significant.

**Figure 3:**
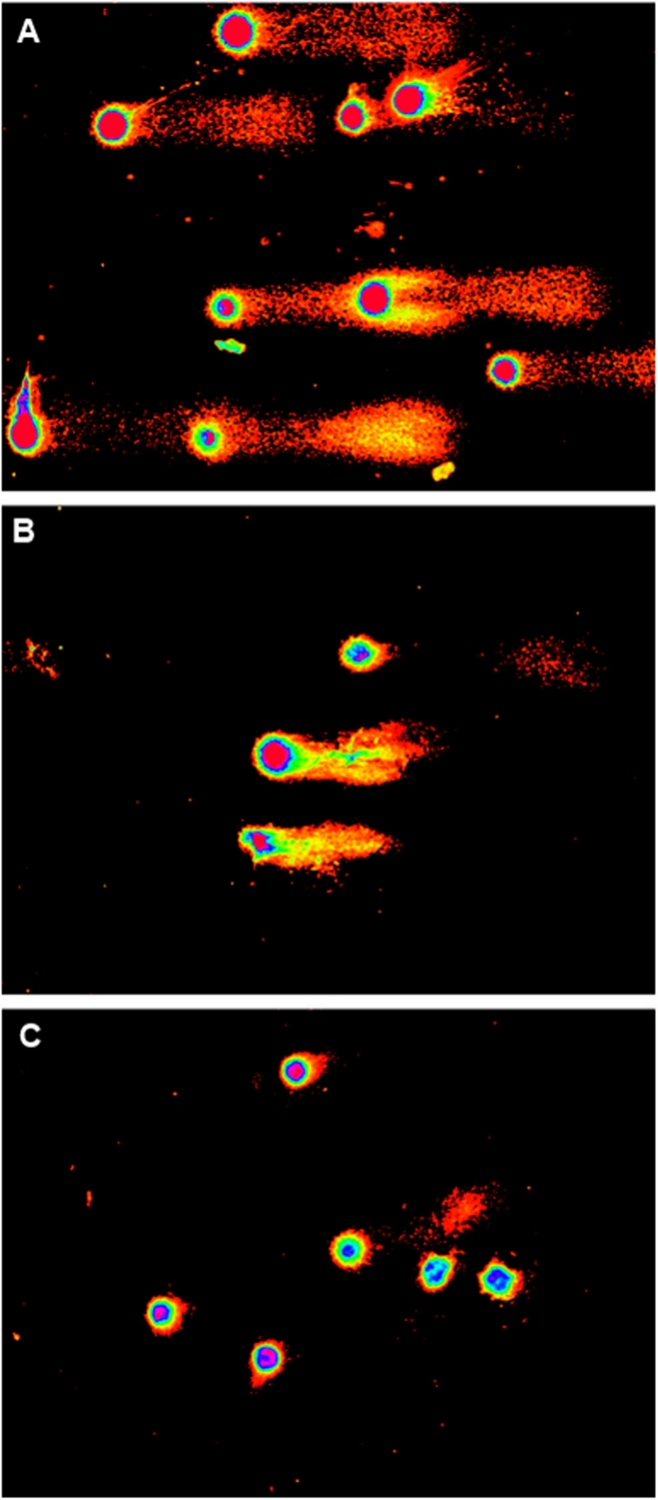
**Comet assay data by using the Comet Score software.** The pink represent the DNA in nucleus, while the orange the fragmented DNA. **A**: Control MCF-7 cell line. **B**: MCF-7 after incubation with doxorubicin. **C**: The same cell line post-incubation with melphalan. The percentage of DNA in tail has been reduced, indicating the effect from the drugs.

Analysis of results was based on percentages of DNA in the comet “head” (amount of genetic material distributed in the nucleus) and in “tail” (amount of genetic material distributed in the fragmented pieces). We examined ≥ 100 cells for each combination of cells and drugs. With *p* set to be 0.05, we estimated the ranges of DNA percentage in untreated and treated cells; where these ranges overlapped, the cells were rejected. In the MCF-7 and MDA-MB 231 cell lines, we clearly observed functional activity for all drugs. The T47D cells showed functional activity for melphalan and doxorubicin, but not for mechlorethamine. All the colorectal cancer cell lines except LoVo, were also successfully affected (Tables 1, 2, 3 and 4; Figures 1, 2 and 3).

**Table 1:** **DNA (%) presented in “head” and “tail” pre- and post-incubation with doxorubicin in breast cancer cell lines**

	Control		Cisplatin (1 μM)	
	% DNA	% DNA	% DNA	% DNA
	(mean ± 1.96 S.E.)	(mean ± 1.96 S.E.)	(mean ± 1.96 S.E.)	(mean ± 1.96 S.E.)
	in Head	in Tail	in Head	in Tail
LoVo	83.55 ± 4.25	17.45 ± 4.12	89.28 ± 2.32	11.72 ± 2.29
HCT-15	78.58 ± 4.05	21.42 ± 4.05	92.35 ± 1.84	7.65 ± 1.83
HCT-116	84.92 ± 3.32	15.08 ± 3.27	92.50 ± 2.00	8.51 ± 1.88
HT55	82.17 ± 2.75	17.82 ± 2.75	90.60 ± 1.58	9.39 ± 1.51

**Table 2:** **DNA (%) presented in “head” and “tail” pre- and post-incubation with melphalan in breast cancer cell lines**

	Control		Melphalan (1 μM )	
	% DNA	% DNA	% DNA	% DNA
	(mean ± 1.96 S.E.)	(mean ± 1.96 S.E.)	(mean ± 1.96 S.E.)	(mean ± 1.96 S.E.)
	in Head	in Tail	in Head	in Tail
MCF-7	66.43 ± 3.68	33.56 ± 3.68	83.96 ± 2.42	16.04 ± 2.35
MDA-MB 231	77.14 ± 2.98	22.85 ± 2.98	82.66 ± 2.33	17.34 ± 2.33
T47D	77.87 ± 2.60	22.12 ± 2.60	85.37 ± 1.93	14.62 ± 1.93

**Table 3:** **DNA (%) presented in “head” and “tail” pre- and post-incubation with mechlorethamine in breast cancer cell lines**

	Control		Mechlorethamine ( 0.1 μM)	
	% DNA	% DNA	% DNA	% DNA
	(mean ± 1.96 S.E.)	(mean ± 1.96 S.E.)	(mean ± 1.96 S.E.)	(mean ± 1.96 S.E.)
	in Head	in Tail	in Head	in Tail
MCF-7	71.03 ± 4.16	28.96 ± 4.16	83.71 ± 2.31	16.64 ± 2.26
MDA-MB 231	71.83 ± 2.49	28.16 ± 2.49	86.72 ± 1.52	13.43 ± 1.49
T47D	84.64 ± 2.77	84.64 ± 2.77	85.75 ± 2.11	14.24 ± 2.11

**Table 4:** **DNA (%) presented in “head” and “ tail ” pre- and post-incubation with doxorubicin in breast cancer cell lines**

	Control		Doxorubicin (1 μM)	
	% DNA	% DNA	% DNA	% DNA
	(mean ± 1.96 S.E.)	(mean ± 1.96 S.E.)	(mean ± 1.96 S.E.)	(mean ± 1.96 S.E.)
	in Head	in Tail	in Head	in Tail
MCF-7	66.43 ± 3.68	33.56 ± 3.68	81.33 ± 2.67	19.17 ± 2.62
MDA-MB 231	77.14 ± 2.98	22.85 ± 2.98	83.93 ± 1.94	16.06 ± 1.94
T47D	77.87 ± 2.60	22.12 ± 2.60	83.17 ± 2.27	16.82 ± 2.27

**Figure 4:**
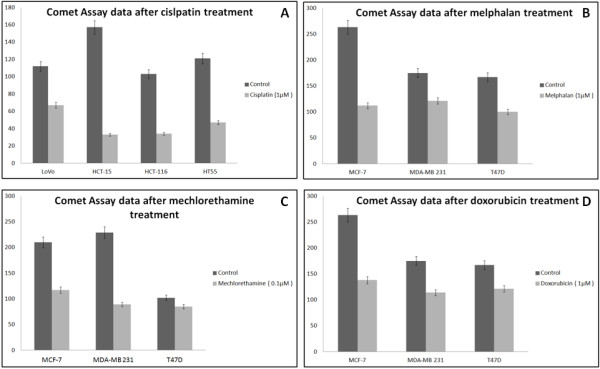
**Presentation of comet assay data by using a five-grade scale.** The comets are classified according to extent of DNA in tail and a value 0-4 is given (0: almost no DNA in tail, 4: almost the entire DNA in tail). By measuring 100 comets, an overall score between 0 and 400 arbitrary units is calculated. **A**: Treatment with cisplatin in colorectal cancer cell lines. **B**: Treatment with melphalan in breast cancer cell lines **C**: Treatment with mechlorethamine in breast cancer cell lines **D**: Treatment with doxorubicin in breast cancer cell lines.

Comet visual scoring can be also calculated based on a five-grade scale from 0 to 4. In a grade 4 comet cell, the entire DNA is in the “tail”, while in grade 0 the entire DNA is in “head”. These values given to comets can be summarized and provide a quantitative measure for 100 cells on a scale from 0-400. These data are presented in Figure 4. A high score means that the cells have been damaged, and the most DNA is in “tail”. The decrease of the score indicates unfragmented cells, therefore a positive effect by the particular drug. The higher the decrease, the greater the effect of the chemotherapeutic. As it is presented in Figure 4, it is displayed little difference in the score of T47D under the treatment with mechlorethamine. It is also observed that among the colorectal cancer cell lines, cisplatin affects HCT-15 more than the others. Among MCF-7, MDA-MB 231 and T47D, the MCF-7 cell line is more affected by melphalan and doxorubicin, while MDA-MB 231 is more sensitive in mechlorethamine.

Breast cancer is the most lethal malignancy in women [7]. Colorectal cancer is the second most common cause of cancer in women, the third most common in men, and the fourth most common cause of cancer death overall [8]. Management of cancer focuses on cure (achievable through surgery, chemotherapy or radiation) or palliation (an important consideration in incurable disease) [9, 10]. Among the variety of chemotherapeutic drugs against breast and colorectal cancer are alkylating agents, which can prevent DNA synthesis and RNA transcription by cross-linking DNA; or by attaching to DNA bases, causing them to be fragmented by repair enzymes. Alkylating agents can also induce mispairing of nucleotides. As the mode of action depends on the genetic structure, a means of detecting changes in DNA structure is needed [11]. Nitrogen mustards are non-specific cytotoxic chemotherapy agents, and include cyclophosphamide, chlorambucil, uramustine, ifosfamide, melphalan, bendamustine and mechlorethamine [12].

Melphalan in combination with other drugs, or as monotherapy, is highly active in patients with advanced breast cancer. It attaches an alkyl group to the 7′ nitrogen atom of the imidazole ring, in the DNA guanine base. Melphalan has several side effects including leucopenia, thrombocytopenia and anemia, nausea, vomiting and diarrhea [13]. Mechlorethamine also known as mustine (HN2) is a derivative of mustard gas. It prevents cell duplication by cross linking DNA; it is cell cycle phase-nonspecific. It is used in humans and other mammals. Overexposure can lead to leukopenia, anemia and thrombocytopenia, while chromosomal abnormalities, hepatotoxicity, neurotoxicity and cardiac irregularities have been reported; both its hematologic and gastrointestinal side effects are usually dose-dependent. [14]. Neurotoxicity in the form of local-regional sensory has been observed especially in combination with cisplatin (CDDP). Cisplatin binds to DNA to cause cross linking, which leads to apoptosis; it is often combined with other chemotherapy drugs in cancer treatment [15]. In breast cancer, an anthracycline antibiotic is also used; it inhibits the enzyme topoisomerase II, thus inhibiting transcription. Doxorubicin (trade name Adriamycin) is often used in chemotherapy combinations such as AC (doxorubicin-cytoxan), ATC (doxorubicin-cyclophosphamide-Taxol), AT (doxorubicin-taxotere), and FAC (fluorouracil-doxorubicin-cytoxan) [16, 17]. Dose-limiting side effects, such as myelosuppression and cardiotoxicity, have been reported after treatment with doxorubicin [18]. Excluding side effects can greatly improve healing of carcinomas. Recent studies have indicated that MAP kinases play an important role in resistance to treatment with doxorubicin, mechlorethamine, paclitaxel and proteasome inhibitors [19].

Many techniques to predict chemotherapy response are available. Assays based on PCR can show genes for enzymes that affect metabolism of these drugs, and can therefore indicate response to a given agent [20, 21]. However, they predict response at a genomic level, which often deviates significantly from protein and cellular levels. On the other hand, the study of a great number of proteins displays many restrictions. First, we need high amounts of proteins, which mean a lot of genetic material. Furthermore, the use of Western Blot technique is intended for a limited number of proteins. Additionally, the response to chemotherapy drugs is affected be many alleles (CYB2D6, CYP2C19, CYP3A4 etc.), so the study of all them in most cases is expensive and time-consuming. Other techniques are based on molecular imaging, such as 18 F-fluorodeoxyglucose-positron emission tomography (18-FDG-PET) or magnetic resonance imaging (MRI) [22], but these methods have common adverse effects. Both techniques are non-invasive, however 18-FDG-PET involves exposure to ionizing radiation and the MRI environment may cause harm in patients by using strong magnetic fields and radiowaves. Comet assay require only a few of a patient’s cells, which can be easily isolated from whole blood. By administrating the chemotherapy agents and then applying an electric field to the DNA, unfragmented DNA moves slowly because it is too large, whereas fragmented parts move faster because of their lighter molecular weight and dense conformation. Unfragmented DNA is thus distributed in the nucleus - the comet’s “head” - and fragmented pieces in the “tail”. The comparative percentage of DNA in a single cell’s “head” and ”tail” reflects the effect of a drug on its DNA chains. These percentages are ascertained from the assignment of the pixels in photographs of the assay, as a percentage with no units [23]. By analyzing the above data from many cells, the effect of a drug can be determined. This method can be used also to study genetic instability, or genotoxicity [24, 25]. Comet assays can be used to determine bladder cancer cell radiation as well as radiosensitivity in other types of cancer [26].

This particular work contains numerous cancer cell lines, which represent two widespread types of cancer. For each cancer type cell lines with different properties were tested. Also there is a study of several most common chemotherapeutic drugs.

The experimental data demonstrated that personalized treatment is essential in cancer treatment. The data from colon cancer cell lines indicated that cisplatin does not affect each cell line to the same grade. Also, in some cases, like LoVo, the cisplatin is probably ineffective. Between HCT-15 and LoVo, which both represent colon adenocarcinoma, completely different data were observed. However, it is remarkable that LoVo derived from a metastatic tumour. Recent literature data, indicate that cisplatin in combination with other agents is more effective than monotherapy in this particular cell line [27].

Among the breast cancer cell lines, the T47D cells carry receptors for a variety of steroids, while MCF-7 cells express oestrogen receptors. The variety between these receptors may explain the different response to chemotherapy. MDA-MB 231 established from a pleural effusion of a 51 year old woman with metastatic breast cancer, indicating that mechlorethamine might be more effective in metastatic breast tumour.

Comet assays are a sensitive means of detecting both single strand and double strand DNA damage, using only a few cells. Our results here reinforced the heterogeneity of cancer by showing how the effectiveness of each drug varied in each cell line, even when derived from the same cancer type. Potentially, comet assays could allow physicians to evaluate patient response in advance, thus avoiding inappropriate therapies with undesired effects. SCGE is a relatively quick versatile, simple-to-perform technique with a few requirements.

## Conclusion

Tailor-made therapy is a promising theme in translational cancer research. However, identifying optimal treatment for an individual patient can be difficult, time-consuming and expensive. Here, we evaluated the effectiveness of comet assays on four commonly used chemotherapeutic drugs, and found that this technique can predict responses of different cancers to the subject drugs and generally to drugs with the same mechanisms of action. Further studies with a wider variety of tumor cell types and drugs are required to establish the clinical utility of comet assays in selecting cancer treatments.

## Methods

### Cell lines

We used human breast cancer cell lines MCF-7, MDA-MB 231 and T47D, and colon cancer cell lines HCT-116, HT55, HCT-15 and LoVo, which came from the European Collection of Cell Cultures (ECACC - HPA cultures, UK). Cells were cultured in 75 cm^2^ flasks (5520200; Orange Scientific) at 37°C in a 5% CO_2_ atmosphere, in the recommended media supplemented with the appropriate amount of heat-inactivated fetal bovine serum (Invitrogen, 10106-169, California) and 2 mM L-glutamine (Sigma, G5792, Germany).

### Drugs

According to literature data, the optimal concentration of each drug varies by cell type and cancer type. Cells were cultured in 12-well plates (3513, Corning) in different concentrations (0.01-50 μM) for each drug. Their viability was analyzed with propidium iodide staining after 24 hrs of incubation (NucleoCounter® NC-100™, Chemometec). Concentrations that were chosen for further experiments were 0.1 μM for mechlorethamine (122564, Sigma-Aldrich), 1 μM for melphalan (M2011, Sigma-Aldrich), 1 μM for doxorubicin (D1515, Sigma-Aldrich) and 1 μM for cisplatin (P4394, Sigma-Aldrich).

### Comet Assay

Cells were divided into 25 cm^2^ flasks (5520100, Orange Scientific), one for each drug and one with no drug added. After 24 hrs of incubation, cells were detached by trypsinization (Trypsin-0.25% EDTA, 25200-072; Invitrogen) and 20000 cells were used for single cell gel electrophoresis with IKZUS comet assay kit (0905-050-K, IKZUS). Alkaline lysis of the cells was performed according to manufacturer’s instructions. Slides were observed under a UV microscope. Data were analyzed with Comet Score software (TriTek Corp., USA).

## Abbreviations

SCGE: Single cell gel electrophoresis
HN2: Mustine
CDDP: Cisplatin
AC: Doxorubicin-Cytoxan
ATC: Doxorubicin-Cyclophosphamide-Taxol
AT: Doxorubicin-Taxotere
FAC: Fluorouracil-Doxorubicin-Cytoxan
18 F-FDG-PET: 18 F-fluorodeoxyglucose-positron emission tomography
MRI: Magnetic resonance imaging
SE: Standard Error.
